# “Metabolic surgery in Asian patients with type 2 diabetes mellitus and body mass index less than 30kg/m2: A systematic review”

**DOI:** 10.1016/j.obpill.2024.100145

**Published:** 2024-10-21

**Authors:** Angel Alois Osorio Manyari, Azucena Lirio Armas Alvarez, Joel Davis Osorio Manyari, Francisco Gonzalez Caballero, Sjaak Pouwels

**Affiliations:** a Service of Surgery, Hospital Don Benito-Villanueva, Ctra. Don Benito-Villanueva s/n, 06400, Don Benito, Villanueva, Spain; b Service of Urology, Hospital Don Benito-Villanueva, Ctra. Don Benito-Villanueva s/n, 06400, Don Benito, Villanueva, Spain; c Service of Surgery, Hospital Can Misses, Carrer de Corona s/n, Ibiza, 07800, Spain; d Department of Surgery, Marien Hospital Herne, University Hospital of Ruhr University Bochum, Herne, NRW, Germany; e Department of Intensive Care Medicine, Elisabeth-Tweesteden Hospital, Tilburg, the Netherlands

**Keywords:** *Metabolic surgery*, *Bariatric surgery*, *Type 2 diabetes*

## Abstract

**Background:**

The effect of metabolic surgery on long-term diabetes remission in Asian patients with a body mass index (BMI) < 30 kg/m^2^ has not been widely reported.

**Methods:**

We conducted a systematic review of the PubMed and Cochrane Library databases from inception to June 2024. All clinical trials and observational studies involving the effect of metabolic surgery in Asian patients with type 2 diabetes mellitus and BMI <30 kg/m^2^ were considered. The quality of the studies was assessed using the Newcastle-Ottawa scale.

**Results:**

Of the 1175 studies screened, 21 studies (11 prospective and 10 retrospective), including 1005 patients, were selected. Only one study had a control group. The longest follow-up was 60 months. The results showed significant improvement in glycated hemoglobin (HbA1c), fasting blood glucose (FBG), 2-h plasma glucose (2hPG), homeostasis model assessment for insulin resistance index (HOMA-IR), fasting C-peptide, triglycerides, total cholesterol, and a reduction in the use of oral hypoglycemic agents/insulin at 12, 24, 36, and 60 months after metabolic surgery. The most common surgical complications observed were anemia (2.1 %–33 %), marginal ulcer (4.2 %–17.3 %), gastrointestinal bleeding (1.9 %–12 %), anastomotic leak (2.1 %–3.5 %), anastomotic stenosis (2.1 %–3.5 %), reoperation (1.18 %), and a mortality rate of zero.

**Conclusions:**

Long-term diabetes remission, along with improvements in HbA1c, 2hPG, FBG, and HOMA-IR, with an acceptable rate of complications, was observed in Asian patients with BMI <30 kg/m^2^ after metabolic surgery. Future research with controlled studies should focus on preoperative patient selection criteria beyond just the BMI cutoff.

## 1 Introduction

Diabetes mellitus is a global health issue. The global diabetes prevalence among individuals aged 20–79-year-olds in 2021 was estimated to be 10.5 % (536.6 million people), with projections indicating an increase to 12.2 % (783.2 million) in 2045. Asia is a major area of the rapidly emerging Type 2 Diabetes Mellitus (T2DM) global epidemic, with China and India the top two epicenters [1]. Currently, more than 230 million Asian individuals are living with diabetes, accounting for approximately 55 % of the world's population with diabetes, and this number is expected to exceed 355 million by 2040. China and India, have the largest number of patients with diabetes globally, with 110 million and 69.2 million people, respectively. The International Diabetes Federation (IDF) reported that China, India, and Indonesia— all Asian countries—have the highest number of diabetes-related deaths [2]. In Asia, the prevalence of diabetes has increased three to sixfold within the last 20–25 years [3].

In Western countries, the relationship between T2DM and obesity is well established, and 90 % of patients with T2DM have overweight or obesity [4]. However, in Asian countries, patients with T2DM typically have a lower body mass index (BMI) compared to Caucasian [5]. The mean BMIs of patients with T2DM are 32.3 kg/m^2^ in the USA, 29.4 kg/m^2^ in the UK, 23.1 kg/m^2^ in Japan, 24.9 kg/m^2^ in Korea, and 24 kg/m^2^ in China [[6], [7], [8]]. In the USA, 72 % of patients with T2DM have a BMI ≥30 kg/m^2^, compared to 56 % in the UK, while in China, only 25 % of patients with T2DM have a BMI >27.5 kg/m^2^ [[6], [7], [8]]. In East Asia, particularly in China, the predominant factors for developing T2DM in individuals with low or normal BMI include weaker pancreatic islet cells compared to other ethnic groups, a greater propensity for central obesity, which leads to higher susceptibility to insulin resistance rather than having global obesity, and the rapid changes in eating habits and lifestyles due to economic development [[9], [10], [11]].

Several small randomized clinical trials (RCTs) and larger observational studies in patients with obesity demonstrated that metabolic/bariatric surgery is superior to medical and lifestyle therapies for the treatment of T2DM. One RCT study reported that metabolic surgery compared with medical/lifestyle intervention provided superior glycemic control after 7–12 years of follow-up [12].

It has been reported that improvements in T2DM following metabolic surgery often occur before any significant weight loss [13]. Metabolic surgery can lead to the resolution of T2DM within a week, prior to the significant weight loss that occurs over several months [14]. Several weight loss-independent mechanisms are involved in glycemic improvement such as changes in gut hormones (GLP-1 and ghrelin), bile acid signaling, intestinal nutrient sensing, and alterations in the gut microbiota [15]. Based on these mechanisms, some studies have demonstrated the short-term efficacy of metabolic surgery on patients with T2DM and without obesity [16]. According to the IDF, the American Society for Metabolic and Bariatric Surgery (ASMBS), the International Federation for the Surgery of Obesity and Metabolic Disorders (IFSO), and the Chinese Society for Metabolic and Bariatric Surgery; metabolic surgery is recommended for patients with T2DM and a BMI >35 kg/m^2^ and should be considered an optional treatment for patients with T2DM and a BMI of 30–35 kg/m^2^. Given that the prevalence of diabetes and cardiovascular disease is higher at a lower BMI in the Asian population compared to the non-Asian population, the BMI threshold should be reduced by 2.5 kg/m^2^, recommending metabolic surgery for Asian patients with T2DM and BMI ≥27.5, particularly in Chinese populations [[17], [18], [19]].

Therefore, the investigation of metabolic surgery for patients with T2DM and a lower BMI is of increasing significance. Some meta-analysis and systematic reviews had assessed the effect of metabolic surgery on patients with T2DM and lower BMI [20,21], but these studies presented some limitations, such as lack of long-term outcome data, the inclusion of mixed populations (Asian and non-Asian), and not strictly excluding patients with a BMI >30 kg/m^2^. Only one meta-analysis has assessed the effect of metabolic surgery on diabetes remission in Asian people with BMI <30 kg/m^2^; however, this review was conducted in 2019, included only 12 studies, and lacked long-term follow-up [13].

In the last five years, some clinical studies with mid-to long-term follow-up (≥2 years) have been published [21]. The purpose of this systematic review was to evaluate the effectiveness of metabolic surgery in Asian people with T2DM and a BMI <30 kg/m^2^, including its long-term effects on diabetes remission.

## 2 Methods

### 2.1 Search strategy

We conducted a systematic review in the PubMed and the Cochrane Library databases from inception to June 2024, following the recommendations of the PRISMA 2020 statement: an updated guideline for reporting systematic reviews [22]. The review used a combination of the following keywords: “bariatric surgery OR metabolic surgery OR obesity surgery OR Roux-en-Y OR gastric banding OR one anastomosis gastric bypass OR single anastomosis gastric bypass OR mini gastric bypass OR biliopancreatic diversion OR gastrojejunostomy OR ileal interposition OR duodenojejunal bypass OR duodenal jejunal exclusion OR gastric bypass OR Roux-en-Y gastrointestinal reconstruction OR jejunoileal bypass” AND “diabetes OR diabetes mellitus OR type 2 diabetes OR T2DM” AND “overweight OR low BMI OR body mass index <30 kg/m^2^ OR nonobese OR normal weight”. In addition, we screened the references of the included studies. Only articles published in English language were included. The review was conducted in two steps: first, we checked the titles and abstract of the studies to exclude inappropriate ones. Second, we reviewed the full text of the remaining studies to determine whether they met the inclusion criteria. Articles that showed only the abstract were included if sufficient data were reported. Duplicate publications were excluded. Data from articles on identical participants were extracted only from the highest quality study.

### 2.2 Inclusion and exclusion criteria

For inclusion in the systematic review, a study had to fulfill the following criteria: 1) Patients with T2DM patients with age older than 18 years; 2) Clinical trial or observational studies; 3) Studies involving any type of metabolic surgery in patients with a BMI <30 kg/m^2^ before surgery; 4) Reporting at least two outcomes, one of them must be diabetes remission; 5) Follow-up period of ≥6 months; 6) Inclusion of Asian population from Japan, Singapore, South Korea, China, Taiwan, Hong Kong, India, Malaysia, or Thailand; and 7) English was the language of publication. The exclusion criteria were: 1) Patients with type 1 diabetes, gestational diabetes, and people under 18 years old; 2) Animal or in vitro studies, unpublished reports, case reports, systematic reviews, meta-analysis, letters to the editor, abstracts with incomplete data, or reviews; 3) Patients with gastrointestinal pathology such as inflammatory bowel disease or tumors before surgery; and 4) Patients who underwent a second bariatric surgery.

### 2.3 Selection of studies and data extraction

Titles and abstracts of screened articles were identified and reviewed independently by 2 investigators to determine whether they met eligibility criteria for inclusion. Discrepancies regarding whether to include or exclude a study were resolved by consensus with the other investigators.

All the data were extracted independently by two authors. The following data were extracted from the included articles: 1) first author, 2) publication year, 3) country, 4) study design, inclusion and exclusion criteria, 5) number of patients, 6) type of surgery, 7) age, 8) time of follow-up, 9) diabetes duration, and 10) outcomes. The following outcomes were analyzed to assess the metabolic status before and after the bariatric surgeries: rate of diabetes remission, the body mass index (BMI), waist circumference (WC), glycated hemoglobin A1c (HbA1c), fasting blood glucose (FBG), 2-h postprandial glucose (2hPG), fasting C-peptide (FCP), homeostatic model of insulin resistance (HOMA-IR), total cholesterol (TC), triglyceride (TG) levels, and use of oral hypoglycemic agents (OHA) or Insulin. Additionally, we assess the percentage of complications, and mortality.

### 2.4 Data synthesis

Categorical variables were summarized as percentages, while continuous variables were summarized as means, standard deviations and ranges. The extracted data were organized into tables, summarizing the main findings from each study. All tabulated data referring to similar values were analyzed cumulatively. We used the formula mg/dL = 18 × mmol/L to convert mmol/L to mg/dL and standardize the variables. We included all studies with different criteria for diabetes remission based on HbA1 levels of <6 %, <6.5 %, or <7 %, depending on the study. We considered the following timepoints: 6, 12, 24, 36 and 60 months to assess different outcomes. Discrepancies between the two reviewers were resolved through discussion and consensus with the other authors.

### 2.5 Risk of bias assessment

The Newcastle-Ottawa Scale is a 9-star scoring system that includes patient selection, comparability, and outcome, and it was used to evaluate the quality and risk of bias in the included studies. The quality of a study was considered GOOD (score: 7–9 points), FAIR (score: 2–6 points), or POOR (score: 0–1 point) [23]. Two investigators performed these procedures independently, and a third assessor was consulted in case of a discrepancy.

## 3 Results

### 3.1 Description of the studies

**Fig. 1:**
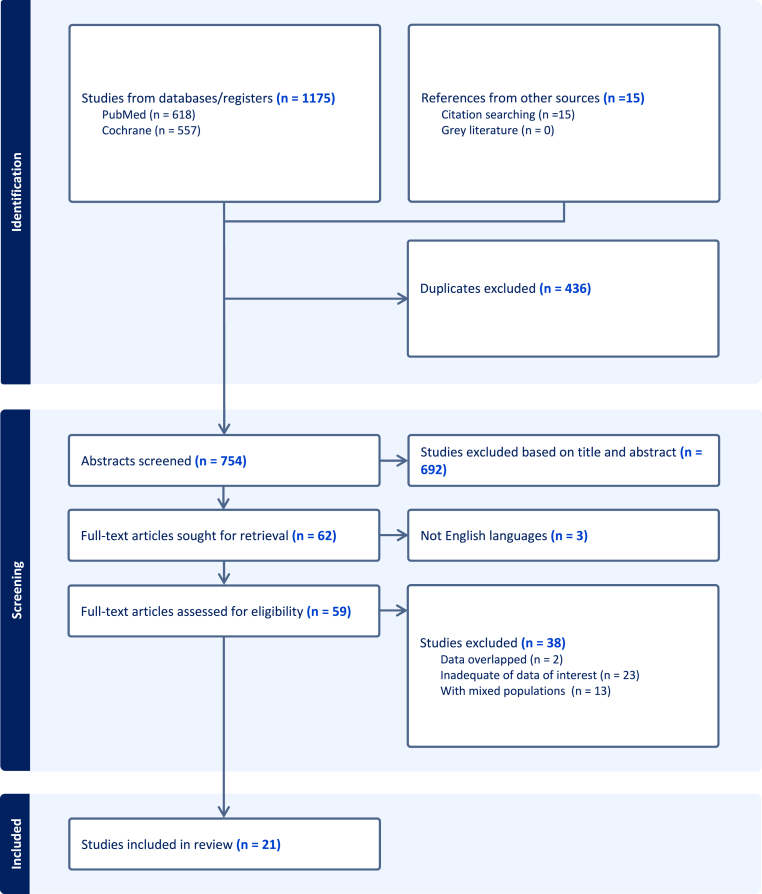
Flow chart of study selection.

A flow diagram of the study selection process is provided in Fig. 1. The literature search identified 1175 potentially relevant articles in the initial electronic search (618 in PubMed and 557 in the Cochrane Library), of which 436 duplicate articles were excluded. After screening the titles and abstracts, we excluded an additional 692 studies based on the inclusion criteria, leaving 62 articles for full-text review. After this review, we excluded 23 studies due to inadequate data of interest, 2 studies due to population overlap, 3 studies because the full publications were not in English, and 13 studies due to mixed populations. In the end, 21 papers were selected for the systematic review.

### 3.2 Population

The overall population of the 21 studies was 1005 patients with T2DM and a BMI<30 kg/m^2^ with a mean age ranging from 45.2 years [14] to 57 years [24]. The most common exclusion criteria reported in the studies were advanced vascular complications, C-peptide <1 ng/mL, addictions to alcohol or drugs, history of mental or physical debilitating diseases, or organ failure. The largest sample size, 172 patients, was reported in a study conducted by Kim et al. [10], while the smallest sample size, 8 patients, was observed in a subgroup analysis reported in another study [25]. Eleven of twenty-one studies were prospectives, and the remaining 10 were retrospective. Regarding the country, 14 studies were from China, 5 from South Korea, 2 from Taiwan, and 1 from India. The follow-up intervals ranged from 6 months [26] to 60 months [27]. In 20 studies, the longest mean diabetes duration was 10 years, with only the study conducted by Cui et al. reporting a more heterogeneous range of diabetes duration, from 1 month to 14 years [28]. The largest prospective study by Kim et al. clearly reported the number of patients during follow-up, starting with 172 patients, reducing to 144 patients at the first year, 116 patients at the second year, and 51 patients at the third year [10]. Other prospective studies reported follow-up rates ranging from 63 % to 84 % at first year [[29], [30], [31]], while the other 8 prospective studies did not provide clear information about follow-up rates (see Table 1).

**Table 1:** Baseline characteristics of studies Abbreviations.

Study/country	Design	Population	Age (years)	Duration T2DM (years)	Intervention	Follow-up (months)
Dixon 2012 [35]/Korea-China	Prospective multicenter study single arm	103	47.5 ± 9.6	8.2 ± 5	LSQGB: 81	12
					LRYGBP: 22	
Zhang 2013 [41]/China	Retrospective observational (subgroup analysis, BMI: 20–30)	G1(BMI 20–25): 10 G2(BMI 25–30): 12	G1: 49.2 ± 4.5 G2:48.5 ± 7.9	G1: 6.9 ± 4.3 G2: 6.7 ± 4.9	LRYGB	12
Heo 2013 [30]/Korea	Prospective Observational study single arm	31	46.6 ± 7.7	8.3 ± 3.7	DYB	12
Malapan 2014 [38]/Taiwan	Clinical trial single arm	29	53 (32–66)	10.4 (2–26)	LRYGB	12
Yin 2014 [32]/China	Prospective single arm (pilot study) (subgroup analysis BMI<27.5)	28	51.6 (27–72)	9.6 (1–28)	LRYGB	15.9 (12–22)
Kim 2014 [10]/South Korea	Prospective single arm	172	46 ± 11	9.6 ± 5.2	LSQGB	1 year: 144
						2 years: 116
						3 years: 51
Chen 2013 [14]/China	Retrospective cohort: 2 arms	76	RYGR: 45.3 ± 8.5	RYGR: 3.7 ± 2.4	SG ​+ ​RYGR: 35 SG ​+ ​B1R: 41	12
			B1R: 49.3 ± 11.5	B1R: 3.1 ± 1.7		
Ramakrishnapillai 2015 [36]/India	Retrospective cohort single arm	G1(BMI 18–24.9):10 G2(BMI 25–29.9):21	45.2 ± 10.2	8.1 ± 4.8	MIHSII	12
Cui 2015 [28]/China	Prospective cohort single arm	58	48.5 ± 12.3	0.1–14	RYGB	12
Liang 2015 [31]/China	Multicenter clinical trial single arm	86	48.5 (22–72)	7 (0.5–17)	RYGB	12
Lee 2015 [29]/Taiwan	Clinical trial single arm (subgroup analysis BMI<30)	80	47.7 ± 9.1	6.5 ± 5.1	LRYGB: 49	12
					LSAGB:21 LSG: 10	
Wang 2016 [40]/China	Retrospective cohort single arm (subgroup analysis, BMI<27.5)	40	49.1 ± 8.1	5.8 ± 2.8	LRYGB	24
Di 2016 [34]/China	Retrospective single cohort	66	50.4 ± 1.4	8.9 ± 5.2	LRYGB	36
Zhang 2016 [37]/China	Retrospective single cohort (subgroup analysis, BMI 25–27.5)	28	51.3 ± 11.1	9.6 ± 5.5	LRYGB	38.7 ± 9.1
Gong 2016 [26]/China	Clinical trial single arm	31	46.2 ± 11.1	8.3 ± 5.7	LRYGB	6
Kwon 2016 [42]/South Korea	Prospective non-randomized study	15	51 (43–55)	10 (7–20)	LRYGB	24
Ke 2017 [39]/China	Prospective uncontrolled clinical trial (subgroup analysis, BMI<30)	47	47.4 ± 8.6		LRYGB	24
Kim 2017 [25]/South Korea	Retrospective cohort (subgroup analysis, BMI<30)	8	51.1 ± 7.1	7.5 ± 2.5	LDYB	36
Wang 2018 [24]/China	Retrospective single cohort	25	57 (24–65)	10 (0–20)	LSG	12
Widjaja 2019 [33]/China	Retrospective single cohort	18	46.3 ± 11.9	2.6 ± 2.2	LSG	12
Wang 2020 [27]/China	Retrospective single cohort (subgroup analysis, BMI<28)	11	52.8 ± 8.8	10.6 ± 3.9	LRYGB	60

Laparoscopic single anastomosis gastric bypass (LSQGB), Laparoscopic Roux-en-Y Gastric bypass (LRYGB), Open approach, Duodenojejunal bypass (DYB), Roux-en-Y Gastric bypass (RYGR). Minimally invasive hybrid surgery for ileal interposition (MIHSII), Laparoscopic sleeve gastrectomy (LSG), Laparoscopic Duodenojejunal bypass (LDYB), Subtotal gastrectomy (SG), Billroth 1 reconstruction (B1R), Roux-en-Y gastrointestinal reconstruction (RYGR), Body Mass Index (BMI) in kg/m^2^, Type 2 diabetes mellitus (T2DM).

### 3.3 Intervention

Different types of surgical procedures were reported: Roux-en-Y gastric bypass (RYGB) in thirteen studies, sleeve gastrectomy (SG) in three, duodenojejunal bypass (DJB) in two, single anastomosis gastric bypass (SAGB) in two studies, Subtotal gastrectomy (SG) with Billroth 1 reconstruction (B1R) in one study and ileal interposition (II) in one study. The laparoscopic was the surgical approach in 18 studies following standardized techniques, while 3 studies reported using the open approach [14,30,31]. Only the study by Chen et al. had a control group [14], while the other 20 studies were single-cohort studies.

### 3.4 Diabetes remission

Totally 20 studies reported the rate of diabetes remission based on HbA1c, but this definition varied across studies; the most frequent adopted definitions were HbA1c < 6 % in 11 studies, HbA1c < 6.5 % in seven studies, and HbA1c < 7 % in two studies [10,32] (see Table 2). At twelve months, the remission rate ranged from 4.8 ​% for SG ​+ ​B1R [14] to 74.2 % for LSG [34]. Considering the type of procedure, diabetes remission rates ranged from 14.2 % [14] to 74.2 % [34] for LRYGB; from 25 % [29] to 78 % [33] for LSG; from 30.1 % [35] to 53 % [10] for LSAGB; 13.3 % for DYB [30]; and 40–57 % for II [36]. Over time the rate of diabetes remission showed some heterogeneous results. It decreased from 74.2 to 57.6 % at 36 months [34], from 71.4 % to 48 % at 36 months [37], and from 63.6 % to 18.1 % at 60 months [27]. On the other hand, Kim et al. [10] reported that remission was sustained and increasing from 53 % to 90 % at 36 months (see Table 2).

**Table 2:** Remissions of type 2 diabetes after surgery.

Author/country	Remission T2DM criteria	Remission (%) 6 m	Remission (%) 12 m	Remission (%) 24 m	Remission (%) 36 m	Remission (%) 60 m
Dixon 2012 [35]/Korea-China	HbA1c < 6.0 % at 12 months		30.1 %			
Zhang 2013 [41]/China	HbA1c < 6.0 %, normal FBG, no diabetic medications at 12 months		LSQGB: 20 %			
			LRYGBP: 25 %			
Heo 2013 [30]/Korea	HbA1c < 6 % without T2DM medication		13.30 %			
Malapan 2014 [38]/Taiwan	HbA1c <6.5 % without oral hypoglycemic agents or insulin		37.9 %			
Yin 2014 [32]/China	HbA1c < 7.0 %.		67.9 %			
Kim 2014 [10]/South Korea	HbA1c < 7.0 %.		53 %	63 %	90 %	
Chen 2013 [14]/China	HbA1c less than 6 % and FBG less than 109 mg% and not antidiabetic medications		RYGR: 14.3 % B1R: 4.8			
Ramakrishnapillai 2015 [36]/India	HbA1c < 6 % in the absence of any diabetic medications for 12 months		G1: 40 %			
			G2: 57 %			
Cui 2015 [28]/China	Fasting blood glucose <126 mg%, 2-h postprandial blood glucose <200 mg%, random blood glucose <200 mg%		82.8 %			
Liang 2015 [31]/China	HbA1c < 6 % and FBG <110 mg/dL without the use of anti-diabetic medication 12 months after surgery		25.1 %			
Lee 2015 [29]/Taiwan	HbA1c < 6.0 % without the use of oral hypoglycemic drugs or insulin		25.0 %			
Wang 2016 [40]/China	HbA1c < 6.5 %, FBG <100 mg%, and 2-h postprandial blood glucose <140 mg% maintained for more than a year without administration of hypoglycemic agents			35 %		
Di 2016 [34]/China	HbA1c level <6.5 % and a fasting glucose concentration <126 mg% without active pharmacologic intervention		74.2 %		57.6 %	
Zhang 2016 [37]/China	HbA1c < 6.0% with a fasting glucose concentration <100 mg% for 1 year or more without active pharmacological intervention.		71.4 %		48 %	
Gong 2016 [26]/China	HbA1c < 6.5% without medication	70 %				
Kwon 2016 [42]/South Korea	HbA1c < 6.5 % without medication			47 %		
Ke 2017 [39]/China	HbA1c < 6 % and fasting glucose level <100 mg% for at least a year without active pharmacologic therapy.		28.2 %			
Kim 2017 [25]/South Korea	HbA1c ≤ 6.5 without oral hypoglycemic agent or insulin.				0	
Wang 2018 [24]/China	HbA1c < 6.5 % or a significant decrease of doses of hypoglycemic drugs after operation	60 %	68 %			
Widjaja 2019 [33]/China	HbA1c levels <6.0 %.		72 %			
Wang 2020 [27]/China	HbA1C ≤ 6.5 % for ≥1 year without active pharmacologic intervention		63.6 %		36.3 %	18.1 %

Abbreviations.

*Hemoglobin A1c (HbA1c), Fasting blood glucose (FBG), Type 2 diabetes mellitus (T2DM), Laparoscopic single anastomosis gastric bypass (LSQGB), Laparoscopic Roux-en-Y Gastric* bypass *(LRYGB), Roux-en-Y Gastric bypass (RYGR), Billroth 1 reconstruction (B1R), G1: BMI 18-24.9, G2: BMI 25-29.9.*

### 3.5 HbA1c

All studies reported HbA1c levels both before and after the procedure. The baseline mean HbA1c ranged from 7.7 % [25] to 10 % (38). A significant reduction in mean HbA1c was observed in 20 studies, with post-procedure HbA1c levels ranging from 4.5 % [28] to 7.2 % [38] after 12 months. However, no significant reduction in HbA1c was found post-DYB in the study by Heo et al. [30] or post-GS ​+ ​B1R in the subgroup analysis by Chen et al. [14]. The reduction in HbA1c was maintained over time post-procedure at 24 and 36 months in 5 studies [10,27,34,37,39]. Wang et al. reported a sustained reduction in HbA1c at 60 months compared to baseline [27], although the HbA1c value was higher compared to the 36-month timepoint (see Table 3).

**Table 3:** Metabolic changes after surgery.

Study/country	HbA1c (%) baseline	HbA1c (%) 6 m	HbA1c (%) 12 m	HbA1c (%) 24 m	HbA1c (%) 36 m	FBG Baseline	FBG 6 m	FBG 12 m	FBG 24 m	FBG 36 m	2hPG baseline	2hPG 6 m	2hPG 12 m	2hPG 24 m	2hPG 36 m
Dixon 2012 [35]/Korea China	9.1 ± 1.6		6.8 ± 1.7^a^												
Zhang 2013 [41]/China	G1: 8.3 ± 1 G2: 8.4 ± 1		G1: 7 ± 0.7^a^ G2: 6.8 ± 1^a^			G1: 171 ± 43 G2: 163 ± 36		G1: 140 ± 37 G2: 133 ± 36^a^			G1: 270 ± 48 G2: 239 ± 72		G1: 171 ± 50^a^ G2: 187 ± 37^a^		
Heo 2013 [30]/Korea	8.2 ± 1.6		7.7 ± 1.9												
Malapan 2014 [38]/Taiwan	10 ± 1.8		7.2 ± 1.4^a^												
Yin 2014 [32]/China	9.3 ± 2.9	6.75 ± 1.06^a^	7.05 ± 1.37^a^			179.2 ± 68.6	123 ± 32.4^a^	128 ± 36^a^							
Kim 2014 [10]/South Korea	9 ± 1.7		6.9 ± 1.5^a^	6.7 ± 1.4^a^	6.2 ± 0.8^a^	188 ± 63		149.2 ± 55.9^a^	145.5 ± 46.4^a^	128.7 ± 67.2^a^	317.2 ± 94.9		201.7 ± 91.6^a^	236.8 ± 80^a^	134.7 ± 24^a^
Chen 2013 [14]/China	RYGR: 9.8 ± 2.3		RYGR: 5.9 ± 0.7^a^			RYGR: 207.2 ± 43.2		RYGR: 135 ± 19.8^a^							
	B1R: 9.3 ± 2.1		B1R: 8.3 ± 1			B1R: 212.6 ± 48.6		B1R:169.9 ± 27.4^a^							
Ramakrishnapillai 2015 [36]/India	8.8		6.8^a^												
Cui 2015 [28]/China	9.1 ± 1.8	5.2 ± 1.3^a^	4.5 ± 1.5^a^			216.2 ± 59.3	111.7 ± 18^a^	106.3 ± 21.6^a^			277.4 ± 81.7	144.5 ± 41.4^a^	138.7 ± 34.3^a^		
Liang 2015 [31]/China	8.5 ± 2.1		6.5 ± 1.4^a^			177.8 ± 81.3		118.4 ± 22.3^a^			334.6 ± 111.7		163.8 ± 48.9^a^		
Lee 2015 [29]/Taiwan	9.1 ± 1.8		6.8 ± 1.3^a^			185.6 ± 39.9		122.1 ± 32.9^a^							
Wang 2016 [40]/China	8.2 ± 1.8	6.8 ± 0.9^a^	7.1 ± 1.3^a^	7.8 ± 2.2^a^		136.9 ± 28.8	109.9 ± 27^a^	120.7 ± 23.4	113.5 ± 21.6^a^		306.8 ± 68.1	266.2 ± 171.4	250.3 ± 50.9^a^	239.3 ± 54^a^	
Di 2016 [34]/China	8.3 ± 1.9	6.1 ± 0.7^a^	6.3 ± 1.1^a^		6.6 ± 1.1^a^	156 ± 55.8	106.3 ± 19.8^a^	108.7 ± 19.8^a^		118.6 ± 32^a^	239.6 ± 73.8	140.6 ± 55.8^a^	138.7 ± 48.5^a^		178 ± 84.6^a^
Zhang 2016 [37]/China	8.5 ± 1.7		6.4 ± 0.8^a^		6.3 ± 0.7^a^	169 ± 66.6		109.9 ± 18.3^a^		108.1 ± 34^a^	261.5 ± 79.4		140.7 ± 45.9^a^		163.4 ± 90^a^
Gong 2016 [26]/China	7.8 ± 1.7	6.5 ± 0.6^a^				201.8 ± 55	124.3 ± 27.9^a^								
Kwon 2016 [42]/S. Korea	8.8 (8.4–9.1)														
Ke 2017 [39]/China	8.2 ± 2.1			6.39 ± 0.8^a^		160.8 ± 59.6			115.2 ± 36.3^a^		300.2 ± 84.6			132.3 ± 60^a^	
Kim 2017 [25]/South Korea	7.7 ± 1.3		7.14^a^	7.34^a^	7.3^a^										
Wang 2018 [24]/China	8.2 ± 1.4	6.5 ± 1^a^	6.5 ± 0.8^a^			185.5 ± 47.9	124 ± 18.6^a^	119.2 ± 17.8^a^							
Widjaja 2019 [33]/China	8.3 ± 1.8	6.2 ± 0.6^a^	5.9 ± 0.7^a^			151.3 ± 55.8	104.4 ± 10.8^a^	100.2 ± 12.5^a^							
Wang 2020 [27]/China	8.5 ± 1.3		6.5 ± 0.9^a^		6.9 ± 1.2^a^	153.3 ± 47.4		108.2 ± 20.9^a^		115.3 ± 25.7^a^	269.3 ± 64.6		138.7 ± 50.6^a^		164.7 ± 54^a^

Abbreviations.

Glycated hemoglobin (HbA1c), 2-h postprandial Blood test (2hPG), Fasting blood glucose (FBG), Roux-en-Y Gastric bypass (RYGR), Billroth 1 reconstruction (B1R), G1: BMI 20–25, G2: BMI 25–30.

a (p < 0.05).

### 3.6 Fasting blood glucose

Only 14 out of 21 studies reported FBG levels pre- and post-procedure. The baseline mean FBG ranged from 136.9 ± 28.8 mg% [40] to 216 ± 59 mg% [28]. A significant reduction in mean FBG was observed in 13 studies after 12 months post-procedure, with FBG levels ranging from 100 ± 12 mg% [33] to 169.3 ± 27 mg% [14]. The reduction in HbA1c was maintained over time post-procedure at 24 and 36 months in five studies [10,27,34,37,39]. Wang et al. [27] reported a sustained reduction in FBG levels post-LRYGB at 60 months compared to baseline, although FBG levels were higher compared to the 36-month time point (see Table 3).

### 3.7 2-h postprandial plasma glucose

Only 9 out of 21 studies reported 2hPG levels pre- and post-procedure. The baseline mean 2hPG ranged from 239.6 ± 73.8 mg% [34] to 334.6 ± 111.7 mg% [31]. The reduction in mean 2hPG was significant in seven studies after 12 months post-procedure, with 2hPG levels ranging from 138.7 ± 34 mg% [28] to 250.2 ± 50.3 mg% [40]. The reduction in mean 2hPG was also significant at the 24-month time point in three studies [10,39,40], and at the 36-month time point in the study conducted by Kim et al. [10]. On the other hand, two studies reported lower values of 2hPG at 36 months compared with baseline but higher than at 12 months post-procedure [34,37]. Wang et al. [27] reported a 2hPG value lower than baseline but higher than at the 12-month time point at 60 months post-LRYGB (see Table 3).

### 3.8 Oral hypoglycemic agents and insulin use

Only 5 studies reported the use of OHA or insulin pre- and post-procedure. The baseline OHA use ranged from 63.6 % [34] to 81.8 % [27]. A significant reduction in OHA use was observed in the five studies, ranging from 3 % [34] to 40 % [30]. This reduction was sustained at 36 months post-procedure in two studies [27,37]. The baseline insulin use ranged from 10.5 % [14] to 71.2 % [34]. A significant reduction in insulin use was reported in the five studies, ranging from 3 % [34] to 22.7 % [30]. This reduction was sustained at 36 months post-procedure in three studies [27,34,37].

### 3.9 BMI

BMI was calculated as weight in kilograms divided by the square of the height in meters. Only 18 out of 21 studies reported changes in BMI before and after metabolic surgeries. The baseline mean BMI was reported to be between 22.3 ± 1.5 kg/m^2^ [14] and 29.3 ± 0.9 kg/m^2^ [33]. The reduction in mean BMI was reported as significant in sixteen studies 12 months post-procedure, with BMI values ranging from 21.2 ± 1.8 kg/m^2^ [24] to 23.9 ± 0.9 kg/m^2^ [33]. Two studies did not find a significant reduction in BMI at 1-year post-DYB [30] and post-RYGB and B1R [14]. The reduction in BMI post-procedure was sustained at 24 months in two studies [10,39] and at 36 months in four studies [10,27,34,37]. In the study by Wang et al. [27], during a follow-up of 60 months, the mean BMI was significantly lower compared with the baseline but slightly higher compared with the 36-month time point (see Table 4).

**Table 4:** BMI and waist circumference changes after Surgery.

Study/country	BMI baseline (kg/m^2^)	BMI 6 m (kg/m^2^)	BMI 12 m (kg/m^2^)	BMI 24 m (kg/m^2^)	BMI 36 m (kg/m^2^)	BMI 60 m (kg/m^2^)	WC (cm) baseline	WC (cm) 12 m	WC (cm) 36 m	WC (cm) 60 m
Dixon 2012 [35]/Korea-China	26.0 ± 3.0									
Heo 2013 [30]/Korea	23.1 ± 1.3		23.4 ± 0.7							
Malapan 2014 [38]/Taiwan	24.4 ± 1.8		20.6 ± 1.7^a^							
Yin 2014 [32]/China	24.7 (19.4–27.3)		20.8 ± 2.1^a^				89 ± 8.1	75.2 ± 7.3^a^		
Kim 2014 [10]/South Korea	25.3 ± 3.2		22.9 ± 3.0^a^	22.5 ± 3.5^a^	22.4 ± 4.1^a^					
Chen 2013 [14]/China	RYGR: 22.3 ± 1.5		RYGR: 20.7 ± 2.1							
	B1R: 22.9 ± 2.1		B1R: 19.8 ± 3.1							
Ramakrishnapillai 2015 [36]/India	26.6 ± 2.6	23.3	21.5^a^							
Cui 2015 [28]/China	23.4 ± 2.8	21.9 ± 2.8^a^	21.7 ± 2.7^a^							
Liang 2015 [31]/China	24.6 (18.6–27.9)		21.7 ± 2.4^a^							
Lee 2015 [29]/Taiwan	26.9 ± 2		22.7 ± 2.5^a^							
Wang 2016 [40]/China	19.3–27.2									
Di 2016 [34]/China	28.2 ± 1.2	22.7 ± 1.6^a^	22.5 ± 1.8^a^		23.0 ± 1.7^a^		97.6 ± 5.4	81.7 ± 6.5^a^	82.5 ± 4.4^a^	
Zhang 2016 [37]/China	27.1 ± 0.6		22.1 ± 1.6^a^		22.7 ± 0.9^a^		99.4 ± 6.1	82.6 ± 6.8^a^	86.7 ± 6.5^a^	
Gong 2016 [26]/China	26.5 ± 0.4	21.9 ± 1.1^a^								
Kwon 2016 [42]/South Korea	26.1 (25–27.3)									
Ke 2017 [39]/China	26.7 ± 2.5	21.9^a^	21.7^a^	21.6^a^						
Kim 2017 [25]/South Korea	27 ± 2.5									
Wang 2018 [24]/China	27.7 ± 1.8	21.3 ± 1.5^a^	21.2 ± 1.8^a^				99.8 ± 5.3	76.9 ± 5.2^a^		
Widjaja 2019 [33]/China	29.3 ± 0.9	24.4 ± 1.0^a^	23.9 ± 0.9^a^							
Wang 2020 [27]/China	27.4 ± 0.3		21.9 ± 1.3^a^		21.5 ± 1.6^a^	22.7 ± 2.1^a^	96 ± 3.6	80.5 ± 5.1^a^	81.7 ± 5.2^a^	84.5 ± 6.3^a^

Abbreviations.

Body Mass Index (BMI), Waist circumference (WC). Roux-en-Y Gastric bypass (RYGR), Billroth 1 reconstruction (B1R), G1: BMI 20–25, G2: BMI 25–30.

a (p < 0.05).

### 3.10 Waist circumference

A total of 5 out 21 studies reported waist circumference, all of which reported marked reduction of waist circumference before and after metabolic surgeries. The baseline mean WC was reported to be between 89 ± 8.1 cm [32] and 99.8 ± 5.3 cm [24]. The reduction in mean WC was reported as significant in all 5 studies at 12 months post-procedure, with WC values ranging from 75.2 ± 7.3 cm [32] to 82.6 ± 6.8 cm [37]. The reduction in WC post-procedure was sustained at 24 months in the study conducted by Di et al. [34], and Zhang et al. [37]. In the study conducted by Wang et al. [27], during a follow-up of 36 and 60 months, the mean WC was significantly lower compared with the baseline (see Table 4).

### 3.11 HOMA-IR

Nine out of 21 studies reported changes in HOMA-IR before and after metabolic surgeries. The baseline mean HOMA-IR ranged from 2 [38] to 7 [37]. A significant reduction in mean HOMA-IR was reported in seven studies 12 months after the procedure, with HOMA-IR values ranging from 1.1 [38] to 3.5 [10]. Two studies did not find a significant reduction in HOMA-IR one year after LRYGB [27,40]. The reduction in HOMA-IR was sustained at 24 months and 36 months post-surgery in three studies [10,34,37].

### 3.12 Fasting C-peptide

Totally 12 out 21 studies reported mean FCP changes pre and post metabolic surgeries. The baseline of mean FCP was reported from 1.3 ± 0.7 [31] to 2.8 ± 1.2 [10]. The reduction of the mean FCP was reported significant in 8 studies after of 12 months post procedure, and the FCP ranged from 1 ± 0.6 [40] to 1.7 ± 0.6 [37], while 3 studies did not find a significant reduction in the FCP at 1 year post surgery [10,31,32]. The reduction of the FCP post procedure was sustained at 36 months post-surgery in 3 studies [27,34,37].

### 3.13 Lipid metabolic parameters

Seven out of 21 studies reported changes in total cholesterol and triglycerides before and after metabolic surgeries. The baseline mean CT ranged from 84.6 ± 18.1 [37] to 204.6 ± 33.5 [38]. Four studies reported a significant improvement in CT at 12 months post-procedure, with values ranging from 72.2 ± 14.4 [34] to 174.6 ± 35.8 [38]. This improvement was sustained in the studies by Di et al. [34] and Zhang [37] et al. at 36 months post-LRYGB. The baseline mean TG ranged from 32.1 ± 22.3 [32] to 256.6 ± 246.4 [29]. Six studies reported a significant improvement in TG at 12 months post-procedure, with values ranging from 18.4 ± 7.1 [37] to 121.3 ± 64.6 [29]. This improvement was sustained in the studies by Di et al. [34] and Zhang et al. [37] at 36 months post-LRYGB.

### 3.14 Surgical complications/mortality

Only 18 out 21 studies reported surgical complications, including: gastrointestinal bleeding in 6 studies, with incidences ranging from 1.9 % to 12 %; marginal ulcer in 6 studies, with incidences ranging from 4.2 % to 17.3 %; anemia in 5 studies, with incidences ranging from 2.1 % to 33 %; anastomotic leak in 3 studies, with incidences ranging from 2.1 % to 3.5 %; anastomotic stenosis in 3 studies, with incidences ranging from 2.1 % to 3.5 %; gastroesophageal reflux in 1 study, with an incidence of 10 %; reoperation due to marginal ulcer in 1 study, with an incidence of 1.18 %; intestinal obstruction in 1 study, with an incidence of 7.1 %; and mortality was reported as zero in all studies.

### 3.15 Assessment of risk of bias in individual studies

The quality assessments of the included studies are shown in Table 5. Only the study by Chen et al. [14] had a control group, and its quality was rated as GOOD. The other 20 studies were single-cohort studies, and their quality was rated as FAIR with some risk of bias. Out of the 21 studies, 20 had a follow-up period of at least 12 months, except for the study by Gong et al. [29], which had a follow-up period of 6 months and thus diminished the quality of this study.

**Table 5:** Quality of the studies included with the Newcastle–Ottawa scale evaluation.

Study/country	Selection	Comparability	Outcome	Total score (assessment of quality)
Dixon 2012 [35]/Korea-China	3	0	3	6(FAIR)
Zhang 2013 [41]/China	2	0	3	5 (FAIR)
Heo 2013 [30]/Korea	2	0	3	5 (FAIR)
Malapan 2014 [38]/Taiwan	2	0	3	5 (FAIR)
Yin 2014 [32]/China	2	0	3	5 (FAIR)
Kim 2014 [10]/South Korea	2	0	3	5 (FAIR)
Chen 2013 [14]/China	3	1	3	7 (GOOD)
Ramakrishnapillai 2015 [36]/India	2	0	3	5 (FAIR)
Cui 2015 [28]/China	2	0	3	5 (FAIR)
Liang 2015 [31]/China	2	0	3	5 (FAIR)
Lee 2015 [29]/Taiwan	2	0	3	5 (FAIR)
Wang 2016 [40]/China	2	0	3	5 (FAIR)
Di 2016 [34]/China	3	0	3	6 (FAIR)
Zhang 2016 [37]/China	2	0	3	5 (FAIR)
Gong 2016 [26]/China	2	0	2	4 (FAIR)
Kwon 2016 [42]/South Korea	2	0	3	5 (FAIR)
Ke 2017 [39]/China	2	0	3	5 (FAIR)
Kim 2017 [25]/South Korea	2	0	3	5 (FAIR)
Wang 2018 [24]/China	2	0	3	5 (FAIR)
Widjaja 2019 [33]/China	2	0	3	5 (FAIR)
Wang 2020 [27]/China	2	0	3	5 (FAIR)

## 4 Discussion

Bariatric surgery in the context of T2DM treatment, to achieve glycemic control is named metabolic surgery. A meta-analysis of Buchwald et al., over 22094 patients with obesity reported that within studies reporting resolution of diabetes, 1417 (76.8 % [metanalytic mean, 76.8 %; 95 % CI, 70.7%–82.9 %]) of 1846 patients experienced complete resolution T2DM after metabolic surgery [43].

The mean BMI of patients with T2DM in China, South Korea, and Japan ranges from 23.1 to 24.9 kg/m^2^. Emerging evidence suggests that diabetes in Asia has unique features compared to Western countries: an earlier onset of diabetes, a lower BMI, and a greater risk of developing complications. Therefore, determining the efficacy and safety of metabolic surgery for the treatment of T2DM in Asian patients with BMI <30 kg/m^2^ is important [44].

In this study, we pooled data from 1005 Asian patients with T2DM and BMI <30 kg/m^2^ across 21 studies. There was a significant improvement in HbA1c, FBG, 2hPG, and HOMA-IR, BMI, WC, fasting C-peptide, TC, TG, and a reduction in the use of OHA/insulin after 12, 24, 36, and 60 months of metabolic surgery. This study had the longest follow-up compared with other systematic reviews. Similar outcomes have been reported by other researchers. Baskota et al., in a meta-analysis of 290 non-obese T2DM patients, reported statistically significant improvements in HbA1c (−1.88 %), FBG, HOMA-IR levels, and BMI (−2.79 kg/m^2^) after 3–24 months of surgery [45]. In 2018, a systematic review conducted by Huang et al., which included 935 Asian and non-Asian patients with T2DM and BMI <30 kg/m^2^, revealed significant improvements in FBG, HbA1c, 2hPG, HOMA-IR, BMI, WC, fasting C-peptide, TG, and TC during a follow-up period ranging from 12 to 42 months [16]. A meta-analysis including 1105 Asian and non-Asian patients with T2DM and a BMI <30 kg/m^2^ suggested an improvement in glycemic parameters, with a reduction of 2.08 % in HbA1c, 55.93 mg/dL in FBG, and 3.57 kg/m^2^ in BMI at 6–42 months after surgery [20]. In 2019, a meta-analysis of 6 prospective and 6 retrospective studies, including 697 Asian patients with a BMI <30 kg/m^2^, showed that bariatric surgery significantly reduced BMI and waist circumference by 2.88 kg/m2 and 12.92 cm, respectively, at.

12 months postoperatively, as well as FPG, 2hPG, HbA1c, TC, and TG. However, no significant changes were found in C-peptide and HOMA-IR at several time points after surgery (6, 12, and 24 months) [13]. A meta-analysis including the longest follow-up bariatric studies in Chinese patients with T2DM and a BMI <35 kg/m^2^ reported significant decreases in BMI, WC, FPG, HbA1c, and TG postoperatively at 12, 36, and 60 months without large rebounds [46]. In 2023, a meta-analysis examining the mid-to long-term effects (≥2-year follow-up) of metabolic surgery on patients with T2DM and BMI <30 kg/m^2^, which included 18 articles involving 548 Asian and non-Asian patients, reported a significant reduction in BMI (−4.133 kg/m^2^), HbA1c (−1.9 %), FBG, and fasting C-peptide [21]. No information was obtained about the improvement in medication use from other systematic reviews or meta-analyses.

Our data were heterogeneous considering the type of procedure, with RYGB and SG being the most common procedures (61 % and 14 %, respectively). The diabetes remission rate, using the criteria HbA1c < 6.5 % for diagnosing remission of T2DM [47], ranged from 4.8 % to 74.2 % at 1-year post-metabolic surgery. The low rate of remission of 4.8 % was reported in the study conducted by Chen et al., in 2013, which involved the subtotal gastrectomy (SG) with Billroth 1 reconstruction (B1R). This procedure is not a malabsorptive technique, as it preserves both the duodenum and jejunum. The rate of diabetes remission in the rest of the studies exceeded 13 %. Additionally, only 2 out of 21 studies (10, 32) used a 7 % cut-off for diabetes remission criteria [47]. Both of these studies, published in 2014, based their findings on an old criterion of “glycemic control”. Remission rates were greater for LRYGB, ranging from 14.2 % to 74.2 %, and for LSG, ranging from 25 % to 78 %. Over time, the rate of diabetes remission decreased from 74.2 % to 57.6 % at 36 months and from 63.6 % to 18.1 % at 60 months. However, the study by Kim et al. reported an improvement in T2DM remission from 53 % to 90 % at 36 months [10]. The heterogeneity of our data is partially explained by the variation in HbA1c criteria for diabetes remission across studies, which ranged from 6 % to 7 %. A meta-analysis by Baskota et al. reported a diabetes remission rate of 42.4 % (HbA1c level <6 %), but the follow-up durations of the included studies were short (<2 years) [45]. Some studies reported a high rate of diabetes remission for LRYGB (77 %) [47], while other studies claimed that the SAGB procedure had the highest remission rate (93.9 %) [21]. The systematic review by Huang et al. reported that diabetes remission rates ranged from 13.3 % to 90.2 %, based on 20 studies involving patients with T2DM and BMI <30 kg/m^2^ [16]. Another meta-analysis performed by Rubio-Almanza et al. [20] reported a diabetes remission rate of 43 % (95 % CI, 34–53 %). More recently, a meta-analysis published in 2023 reported a diabetes remission rate of 47.5 % after metabolic surgery [21]. The differences in remission rates can be attributed to variations in patient selection, types of surgical procedures, criteria for remission, and follow-up duration among these studies.

The most common surgical complications observed in our study were as follows: anemia (2.1 %–33 %), marginal ulcers (4.2 %–17.3 %), gastrointestinal bleeding (1.9 %–12 %), anastomotic leaks (2.1 %–3.5 %), anastomotic stenosis (2.1 %–3.5 %), reoperations (1.18 %), and a mortality rate of 0 %. A meta-analysis by Baskota [45] reported an overall major complication rate of 6.2 %, a reoperation rate of 1.7 %, and no deaths. Another meta-analysis conducted by Huang et al. [16] identified the following surgical complications: gastrointestinal bleeding (1 %–10 %) and anemia (8 %–33 %). Late complications, including stenosis, intestinal obstruction, diarrhea, and anemia, were reported in a recent meta-analysis by Zou et al. [21]. The same study indicated that 57.1 % of patients with a preoperative BMI <25 kg/m^2^achieved an underweight status (BMI <18.5 kg/m^2^) after duodenal-jejunal bypass-sleeve gastrectomy [48]. While weight loss is often anticipated after surgery, being underweight is a concerning health condition. Therefore, surgeons should be cautious when recommending metabolic surgery for T2DM patients with a BMI below 25 kg/m^2^.

## 5 Limitations

Strengths of our study include: 95 % of the studies included sample sizes larger than 10 patients; nearly 50 % of the studies had a follow-up duration greater than 2 years; 95 % of the studies had at least 1 year of follow-up post-surgery and 1 study had a follow-up of 5 years; and our population was homogeneous, as we included only Asian patients with BMI <30 kg/m^2^. However, this study has some limitations: the criteria for diabetes remission varied among the published studies, with HbA1c cut-off values ranging from 6 % to 7 %. This variation contributed to the heterogeneity in the remission rates of diabetes across the studies. Other limitations were: 47 % of the studies were retrospective, and 95 % were not controlled; we screened only reports written in English, which could introduce reporting bias; and finally, the effects of different types of surgery were not analyzed in sub-groups, only 2 out of 21 studies (9.5 %) were multicenter, and only 1 study (4.8 %) had a control group. Therefore, further controlled multicenter studies are needed to confirm our findings and enhance the external validity of our results.

The reporting of surgical complications was imprecise in most studies, and the timeline of the operation was often not provided. This limitation makes it difficult to fully understand the true scope of the risk-benefit ratio of metabolic procedures.

The Asian American racial group is defined as people having origins in either East Asia, Southeast Asia, or the Indian subcontinent, for what it is heterogenous in nature, comprising populations from China, India, Japan, Korea, the Philippines, and Vietnam. Asian people have been shown to gain weight centrally, at a faster rate than other ethnic groups. Although exists disparities between the subgroups of Asian American people in terms of risk of comorbidities, most studies still group them into one large category, potentially missing important heterogeneity in disease burden and risk. The present study did not consider subgroup analysis of Asian population, because still the exact risk by races has not been determined [48].

Patients with abdominal obesity are at increased risk for heart disease, diabetes, hypertension, dyslipidemia, steatohepatitis and have higher overall mortality rates [49].

Waist circumference is a measurement of abdominal obesity and is used with BMI for identifying adults at increased risk for morbidity and mortality, particularly in the BMI range 25–35 kg/m2. There is population variability in WC values that predict increased risk. Japanese Americans and Indians from South Asia have more visceral fat and therefore and a higher risk of T2DM for a lower BMI and WC compared with White individuals [50]. A WC ≥ 80 cm in Asian females and ≥90 cm in Asian males is considered abnormal. For the before mentioned, WC is a complementary measurement of risk together with BMI in Asian people. However, a limitation of this review is the lack of reporting of WC in most of the studies included.

Measurement of the waist-to-hip ratio provides no advantage over waist circumference alone and is infrequently used by clinicians. It is not recommended as part of the routine obesity evaluation [51].

Recently, the IDF, ASMBS, IFSO, and the Chinese Society for Metabolic and Bariatric Surgery have suggested considering metabolic surgery for patients with a BMI >27.5 kg/m^2^ [[17], [18], [19]]. The next step is to define additional eligibility criteria for metabolic surgery beyond BMI alone to personalize surgical indications and achieve the best results with minimal harm. In 2015, Lee et al. developed the 'ABCD' scoring system, which has been validated to predict T2DM remission based on four preoperative variables: A for age, B for BMI, C for C-peptide, and D for duration of T2DM [52].

## 6 Conclusion

Our systematic review demonstrated that Asian patients with BMI <30 kg/m^2^ can achieve significant diabetes remission, as well as improvements in HbA1c, FBG, 2hPG, and HOMA-IR, BMI, WC, fasting C-peptide, TC, TG, and the use of OHA/insulin. These effects were sustained over time at 12, 24, 36, and 60 months after metabolic surgery, particularly with LRYGB and SG. Although our data showed a relatively low incidence of surgical complications and no mortality, caution is advised when recommending metabolic surgery for patients with a BMI <25 kg/m^2^.

Box 1 **Take away messages**

➢ This systematic review suggests that metabolic surgery may offer potential benefits for the Asian population with T2DM and a BMI <30 kg/m^2^.
➢ To confirm these findings, further long-term clinical trials are needed, specifically designed to test different subgroups within the Asian population.
➢ These studies should also include waist circumference as a marker for morbidity and mortality risk, while clearly reporting any long-term side effects. Furthermore, patient selection should be based on criteria like the ABC score, rather than relying only on BMI cutoffs.

Alt-text: Box 1

## Author contribution

Conceptualization and methodology: OA, SP, AA, OJ. Screening abstracts and full articles: OA, AA, OJ, GF. Investigation, data curation and interpretation of data: OA, AA, OJ, GF. Statistical analysis: OA, AA, OJ, SP. Drafting of the manuscript: OA, AA, OJ, SP. Supervision: OA, SP. Revision, edition and approval of the final submission and publication: OA, AA, OJ, SP.

## Ethical review

The submission represents original work with properly cited sources. The submission does not involve any human test subjects or volunteers.

## Declaration of artificial intelligence (AI) and AI-assisted technologies

During the preparation of this work the authors did not use AI.

## Source of funding

This investigation did not receive any specific grant from funding agencies in the public, commercial, or not-for-profit sectors.

## Declaration of competing interest

The authors declare that they have no known competing financial interests or personal relationships that could have appeared to influence the work reported in this paper.
